# Dynamism of PI4-Phosphate during Interactions with Human Erythrocytes in *Entamoeba histolytica*

**DOI:** 10.3390/microorganisms8071050

**Published:** 2020-07-15

**Authors:** Natsuki Watanabe, Kumiko Nakada-Tsukui, Tomohiko Maehama, Tomoyoshi Nozaki

**Affiliations:** 1 Department of Biomedical Chemistry, Graduate School of Medicine, The University of Tokyo, Tokyo 113-0033, Japan; imm.natsu@gmail.com; 2 Department of Parasitology, National Institute of Infectious Diseases, Tokyo 162-8640, Japan; kumiko@nih.go.jp; 3 Division of Molecular and Cellular Biology, Graduate School of Medicine, Kobe University, Kobe 650-0017, Japan; tmaehama@med.kobe-u.ac.jp

**Keywords:** phosphatidylinositol phosphate, phosphatidylinositol 4-phosphate, pathogenesis, erythrophagocytosis

## Abstract

Phosphatidylinositol phosphates (PIPs) are involved in many cellular events as important secondary messengers. In *Entamoeba histolytica*, a human intestinal protozoan parasite, virulence-associated mechanisms such as cell motility, vesicular traffic, trogo- and phagocytosis are regulated by PIPs. It has been well established that PI3P, PI4P, and PI(3,4,5)P_3_ play specific roles during amoebic trogo- and phagocytosis. In the present study, we demonstrated the nuclear localization of PI4P in *E. histolytica* trophozoites in steady state with immunofluorescence imaging and immunoelectron microscopy, using anti-PI4P antibodies and PI4P biosensors [substrate of the Icm/ Dot type IV secretion system (SidM)]. We further showed that the nuclear PI4P decreased after a co-culture with human erythrocytes or Chinese hamster ovary (CHO) cells. However, concomitant changes in the localization and the amount of PI(4,5)P_2_, which is the expected major metabolized (phosphorylated) product of PI4P, were not observed. This phenomenon was specifically caused by whole or ghost erythrocytes and CHO cells, but not artificial beads. The amount of PIP_2_ and PIP, biochemically estimated by [^32^P]-phosphate metabolic labeling and thin layer chromatography, was decreased upon erythrocyte adherence. Altogether, our data indicate for the first time in eukaryotes that erythrocyte attachment leads to the metabolism of nuclear PIPs, and metabolites other than PI(4,5)P_2_ may be involved in the regulation of downstream cellular events such as cytoskeleton rearrangement or transcriptional regulation.

## 1. Introduction

Phosphatidylinositol phosphates (PIPs) are a class of phospholipids present on plasma and organelle membranes. Seven PIP species, with a combination of 3′, 4′ and 5′ phosphorylations of the inositol ring, are produced and metabolized through the phosphorylation and dephosphorylation by specific kinases and phosphatases in a spatiotemporal fashion. PIPs are known to be involved in a variety of cellular processes including signal transduction, membrane trafficking, and cell migration [1]. During endocytosis, in particular phagocytosis, PI(4,5)P_2_ and PI(3,4,5)P_3_ have been shown to be involved in the recognition of prey, while PI3P is involved in the subsequent maturation of phagosomes in macrophages.

*Entamoeba histolytica* is a parasitic protist and a professional phagocyte, responsible for amebic dysentery and amebic liver abscesses. *E. histolytica* is a major cause of morbidity and mortality in developing countries [2]. The ingestion of microbes and human cells in the colon during commensal colonization and tissue invasion is important for the ameba to acquire nutrients and evade the host immune system. *E. histolytica* exploits two types of ingestion: trogocytosis (the nibbling or chewing of a part of live human cells) and phagocytosis (the internalization of dead cells or undeformable prey, such as bacteria). The significance of PIPs, particularly PI3P and PI(3,4,5)P_3_, in the trogo- and phagocytosis of *E. histolytica* has been well documented [3,4,5]. It has been shown that PI3P, monitored by Hrs-FYVE (Fab-1, YGL023, Vps27, EEA1) and sorting nexin 1 (SNX1) as its biosensor, is recruited to the bottom of the trogo- and phagocytic cups, and subsequently to trogo- and phagosomes [3,6]. On the other hand, the *E. histolytica* FYVE domain containing protein 4 (FP4), which binds to PI4P, has been shown to be recruited to the neck of the closing trogocytic cup and closed (mature) trogosomes [3]. Furthermore, it has recently been reported that PI(3,4,5)P_3_ on the plasma membrane, the trogo- and phagosomes, and the trogo- and phagocytic tunnels/cups, plays a pivotal role in trogo- and phagocytosis, as indicated by the live imaging of the PI(3,4,5)P_3_ effectors, protein kinase A, G, and C families (AGC kinases) [5]. While the spaciotemporal regulation of PIPs and their binding proteins during trogo- and phagocytosis have been unraveled, the roles of PIPs in other cellular compartments such as the ER, Golgi apparatus, and the nucleus remain elusive. In this work, we report for the first time the unprecedented localization of PI4P in the nucleus and the dynamism of PI4P during phagocytosis by immunofluorescence analysis and immunoelectron microscopy using anti-PI4P antibodies and a PI4P biosensors and by the biochemical quantitation of PIPs.

## 2. Materials and Methods

### 2.1. Reagents

All reagents were purchased from Sigma-Aldrich (Missouri, USA) unless otherwise stated. Anti-PI4P (Z-P004) and anti-PI(4,5)P_2_ (Z-G045) antibodies were purchased from Echelon Biosciences (Salt Lake City, USA).

### 2.2. Cell Culture and Human Erythrocytes

Trophozoites of the *E. histolytica* strain HM-1:IMSS cl-6 [7] were maintained axenically in Diamond’s BI-S-33 medium [7] at 35.5 °C. Human erythrocytes were provided by the Japanese Red Cross Society (Tokyo, Japan).

### 2.3. Plasmid Construction

Standard techniques were used for DNA manipulation, subcloning, and plasmid construction. To produce plasmids expressing amino-terminal myc (avian myelocytomatosis virus)-tagged SidM in *E. histolytica* trophozoites, an *E. histolytica*-codon optimized SidM (DQ845395) gene that contained engineered XhoI and XmaI sites on opposite ends was synthesized commercially (GenScript Japan, Tokyo, Japan). The gene fragment was digested with XhoI and XmaI, and cloned into XhoI- and XmaI-double digested pEhEx-myc [8] to yield pmyc-SidM.

### 2.4. Transfection of E. histolytica Trophozoites

The plasmids described above were introduced to amebic trophozoites by lipofection as previously established [9]. Geneticin was added at a concentration of 1 µg/mL at 24 h after transfection and gradually increased for ~2 weeks until the geneticin concentration reached 10 µg/mL.

### 2.5. Immunoblot Analysis

Approximately 1 × 10^5^ of the trophozoites were harvested in the exponential growth phase, and were washed twice with phosphate buffer saline (PBS), pH 7.4, and resuspended in 50 µL of lysis buffer (50 mM Tris-HCl, pH 7.5, 150 mM NaCl, 1% Triton X-100) containing 50 µg/mL of E-64 and cOmplete Mini tablets. Approximately 20 µg of the total cell lysates were separated on 12% sodium dodecyl sulfate (SDS) -polyacrylamide gels and subsequently electrotransferred onto nitrocellulose membranes. The membranes were incubated with 5% non-fat dried milk in TBS-T (50 mM Tri-HCl, pH8.0, 150 mM NaCl and 0.05% Tween-20) for 30 min. The proteins were reacted with primary mouse antibodies specific for myc (with the dilution of 1:1000) or CS1 (1:1000) at 4 °C overnight. After the reaction with primary antibodies, the membranes were washed with TBS-T three times, and further reacted with HRP-conjugated anti-mouse or anti-rabbit IgG antiserum (1:6000 or 1:8000, respectively) at room temperature for 1 h. After washing with the TBS-T three times, the specific proteins were visualized with a chemiluminescence detection using Immobilon Western Chemiluminescent HRP Substrate (Millipore Corporation, Massachusetts, USA) according to the manufacturer’s protocol.

### 2.6. Immunofluorescence Assay (IFA)

A PKH26 prestaining of 1.5 × 10^7^ human erythrocytes was performed by reacting with 1:1000 PKH26 in PBS for 30 min at RT. Then, approximately 5 × 10^3^ *E. histolytica* trophozoites were mixed with the PKH26-labeled erythrocytes in a 50 µL BI-S-33 medium, and co-incubated in 8 mm round wells on a slide glass at 35.5 °C for 15 min. The medium was discarded and the cells were fixed with 660 mM formaldehyde and 100 mM glycine for 10 min. After the cells were washed with PBS and permeabilized with 0.1% digitonin in PBS, containing 100 mM glycine and 0.1% BSA, for 10 min, the cells were blocked with 1% BSA in PBS for 1 h at RT. After the blocking solution was removed, the PBS containing the primary antibodies and 1% BSA was added to the slide glass and the mixture was incubated for 1 h at RT (for the anti-myc antibody (9E10)) or overnight at 4 °C (for anti-PI4P (Z-P004) and anti-PI(4,5)P_2_ (Z-G045) antibodies). After washing twice with 0.1% BSA in PBS, the cells were incubated with anti-mouse IgG Alexa488 labeled antibodies and DAPI in 1% BSA in PBS for 1 h at RT. The antibody dilutions used were: anti-myc mouse IgG at 1:1000, anti PI4P mouse IgM at 1:100, anti-mouse IgM Alexa 488 conjugate at 1:500, and anti-mouse IgG Alexa 488 conjugate at 1:1000. The samples were observed using a Carl-Zeiss LSM 780 Meta laser-scanning confocal microscope. The resultant images were further analyzed using ZEN software (Carl-Zeiss, Oberkochen, Germany).

### 2.7. Quantification of the PI4P Signal Intensity

The fluorescence intensity of the region of the cell with a PI4P signal in the trophozoites stained with the anti-PI4P or anti-myc antibody was captured on a Carl-Zeiss LSM 780 confocal laser-scanning microscope, and the images were analyzed with ZEN software and Adobe Photoshop (Adobe Systems Incorporated, California, CA, USA). The nuclear region was defined by staining with DAPI. The intensity of the PI4P signal in the nucleus, detected with either anti-PI4P or anti-myc antibodies, was expressed as the average fluorescence intensity per pixel after subtracting the intrinsic background signal without staining. The PI4P signal in the cytosol (excluding the nucleus or nuclei) was also measured and expressed as above. The nuclear fluorescence signal was finally defined as the nuclear signal per pixel subtracted by the cytosolic signal per pixel.

### 2.8. Metabolic Labeling of E. histolytica Trophozoites with ^32^P and Co-Incubation with Erythrocyte Membrane Fraction

Approximately 4 × 10^6^ cells of amebic trophozoites were cultured with 250 µCi [^32^P] orthophosphoric acid in a well containing 2 mL of a BI-S-33 medium on a 6-well plastic plate for 4 h at 35.5 °C. After incubation, trophozoites were washed three times with PBS, and co-cultured with the membrane fraction of human erythrocytes for 15 min at 35.5 °C. The membrane fraction was collected from 4 × 10^7^–10^9^ human erythrocytes after a hypoosmotic shock by adding 100 times the volume of distilled water followed by centrifugation at 100,000× *g* at 4 °C for 60 min.

### 2.9. Lipid Extraction

The lipids were extracted by the Bligh−Dyer method. Briefly, approximately 4 × 10^6 32^P of the labeled trophozoites that had been cultured with or without the membrane fraction from 4 × 10^7^–10^9^ human erythrocytes were harvested by a centrifugation at 800× *g* at 4 °C for 3 min. After removing the supernatant, 930 µL of methanol/chloroform/1% perchloric acid (50:25:18, *v*/*v*) was added to the pellet (approximately 70 µL). After vortexing and standing at room temperature for 10 min, 500 µL of chloroform and 500 µL of 1% perchloric acid were added to the mixture. Then, the mixture was vortexed and centrifuged at 1600× *g* at 4 °C for 3 min. The lower organic phase was transferred to a new tube and 1 mL of 1% perchloric acid containing 1 M NaCl was added to the tube. The mixture was briefly vortexed, centrifuged at 1600× *g* for 3 min at room temperature, and the lower phase was transferred to a new tube. After these washing steps were repeated twice, the organic solvents in the lower phase were evaporated by a vacuum evaporator at room temperature for 30 min.

### 2.10. Analysis of Phosphatidylinositol Phosphates by Thin Layer Chromatography

The extracted lipids, prepared as described above, were dissolved in chloroform/methanol (5:1) and applied at the bottom of silica gel 60 plates (TLC Silica gel 60, MERCK) with PIP standards: PI4P (P-9638), PI(4,5)P_2_ (524644, Merck, NJ, USA). The plates were developed using a solution which contained 1.2 g of potassium oxalate, 40 mL of methanol, and 60 mL of deionized water. After development, the plates were baked at 110 °C for 20 min. The solvent (60 mL of chloroform) was drawn up the plate via capillary action. Spots were visualized by incubating the TLC plates in a 5 L saturated tank containing 15 mL of methanol, 46 mL of chloroform, 17 mL of acetone, 14 mL of acetic acid, and 8 mL of distilled water for 3 h. The silica plate was exposed to an imaging plate (Fujifilm, Tokyo, Japan) and the signal was detected by a Typhoon FLA 7000 (GE Healthcare, Chicago, IL, USA). The intensity of the signals was quantified using Fiji (https://imagej.net/Fiji). The signal intensities of individual bands and whole lanes were measured. The signal intensity of each band was normalized against that of the whole lane and expressed relative to the control.

### 2.11. Immunoelectron Microscopic Analysis of PI4P

The sample preparation was carried out as previously described [10,11] with some modifications. Here, the disks with attached amebae were stained with the mouse anti-PI4P monoclonal antibody. A particle density distribution analysis was performed using 24 or 21 immunoelectron micrographs from the erythrocytes (-) and erythrocytes (+) conditions, respectively. The number of anti-PI4P gold particles on the nucleus was counted. The statistical significance between the mean values was determined using Student’s t-test.

## 3. Results

### 3.1. Determination of PI4P Localization by Anti-PI4P Antibody and PI4P Biosensor, SidM, in Steady State and during Phagocytosis of Human Erythrocytes

**Figure 1:**
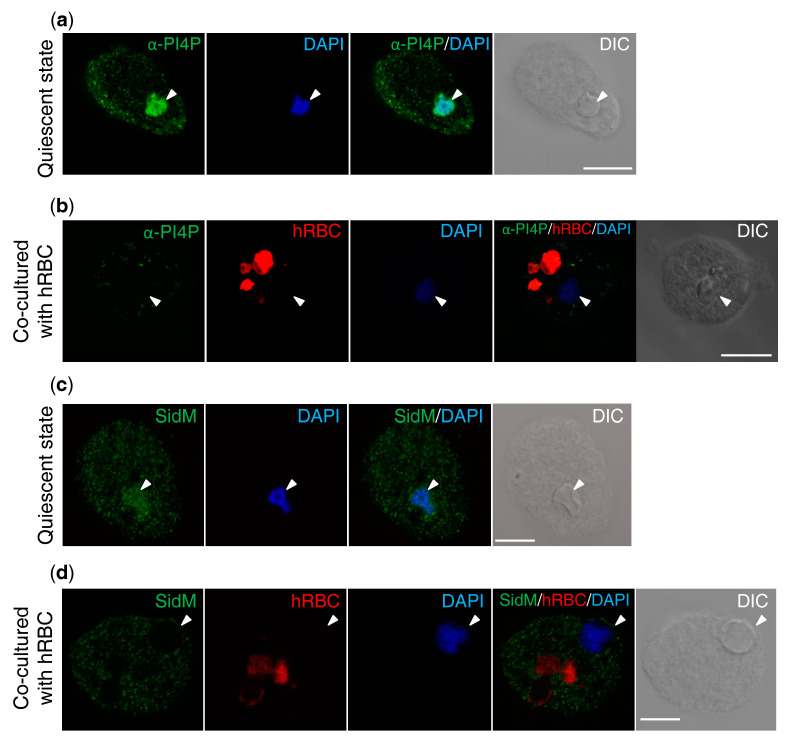
Localization of PI4P in *E. histolytica* trophozoites. (**a**,**b**) Localization of PI4P in steady (quiescent) state (**a**) and during the phagocytosis of human erythrocytes (hRBC) (**b**), demonstrated by the IFA using the anti-PI4P antibody. Note that the PI4P present in both the amebae and hRBCs was detected by the anti-PI4P antibody. (**c**,**d**) Localization of the myc-tagged SidM in the *E. histolytica* transformant strain. The biomarker for PI4P, SidM, fused to the myc tag, was visualized by IFA using anti-myc antibody in steady state (**c**) and during erythrophagocytosis (**d**). Amebic trophozoites were mixed with human erythrocytes which were prestained with PKH26 at RT for 30 min. Nuclei in the amebae and mammalian cells were stained with DAPI after fixation. Arrowheads indicate nuclei. Scale bar = 10 µm.

To better understand the role of PI4P in *E. histolytica* trophozoites, its intracellular distribution was examined by two probes: a PI4P-specific antibody and a PI4P-specific biosensor (SidM domain) [12]. Our previous study showed that EhFP4, which preferentially binds to PI4P (also weakly to PI3P, PI5P, and PS) immobilized on the membrane, was localized to the tunnel-like structures and to the proximal region of the trogosomes in *E. histolytica* during trogocytosis [3]. Based on these observations, we expected PI4P to be localized to the trogosome and involved in trogocytosis. First, PI4P was visualized in the *E. histolytica* wild type strain by an IFA using anti-PI4P antibody. In all the amebic trophozoites, PI4P was localized to punctate structures in the cytosol in steady state. PI4P was also localized in the nucleus in 80–89% (*n* = 3) of trophozoites (Figure 1a). After the addition of human erythrocytes, the PI4P signal in the nuclei greatly diminished (Figure 1b). PI4P was also visualized using an *E. histolytica* transformant strain expressing myc-tagged SidM (Supplementary Figure S1), in which myc-SidM was detected by the anti-myc antibody. The localization of myc-SidM was comparable to that of the PI4P detected with the anti-PI4P antibody; the myc-SidM, detected with the anti-myc antibody, was localized to both the nucleus and the punctate structures in the cytosol in steady state (Figure 1c). No signal was detected in the nucleus after the addition of erythrocytes (Figure 1d), validating the observation of the dynamism of PI4P by the anti-PI4P antibody.

### 3.2. Quantitation of PI4P in the Nucleus in Steady State and during the Phagocytosis of Human Erythrocytes

**Figure 2:**
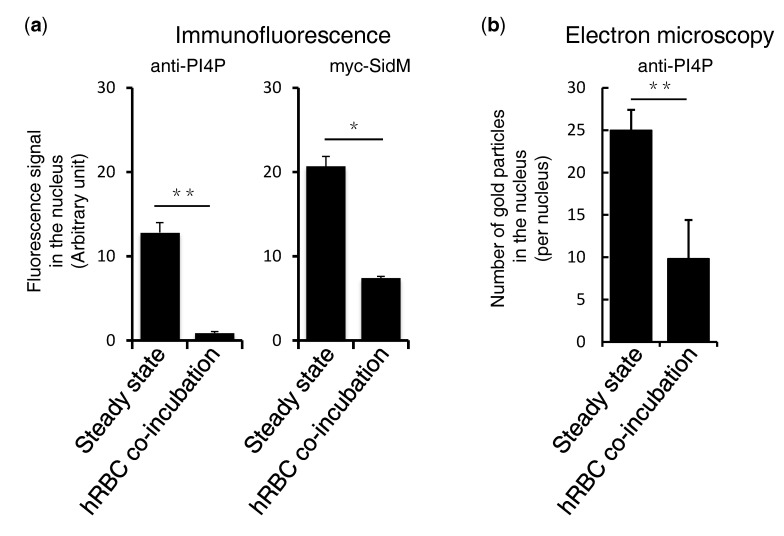
Quantitation of PI4P in the nucleus. Nuclear localized PI4P was quantified by IFA in the HM1:IMSS cl6 wildtype *E. histolytica* cells, using anti-PI4P antibodies, as well as in the myc-SidM expressing transformant cells using anti-myc antibodies (**a**) and by immunoelectron microscopy using anti-PI4P antibodies (**b**). (**a**) Images of ten trophozoites were captured and analyzed using a Carl-Zeiss LSM780 and ZEN software, respectively (*n* = 10). (**b**) The gold particles on the nuclei of 21–24 trophozoites, each for both steady state and co-incubated conditions, were counted. Bars indicate standard errors of replicates. Statistically significant differences with *p* < 0.05 or *p* < 0.01 by Student’s t-test are depicted with “*” or “**”, respectively.

To further investigate the dynamism of PI4P, amebic trophozoites were co-cultured with erythrocytes and PI4P was detected by anti-PI4P antibodies in the wild type cells or anti-myc antibodies in the myc-SidM expressing cells. The images were captured by a laser-scanning microscope before and after the addition of erythrocytes and was analyzed by the methods described in the Materials and Methods section. The observation above, that the nuclear PI4P intensity decreased after the addition of erythrocytes, was reconfirmed by quantitation using either anti-PI4P antibodies in the wildtype cells or anti-myc antibodies in the myc-SidM expressing cells (Figure 2a). PI4P localization was further examined by immunoelectron microscopy using the wildtype cells reacted with anti-PI4P antibodies (Supplementary Figure S2). The number of gold particles in the nuclei detected by the anti-PI4P antibodies significantly decreased after the addition of erythrocytes (Figure 2b).

### 3.3. Elimination of PI4P from Amebic Nuclei is Prey-Dependent

**Figure 3:**
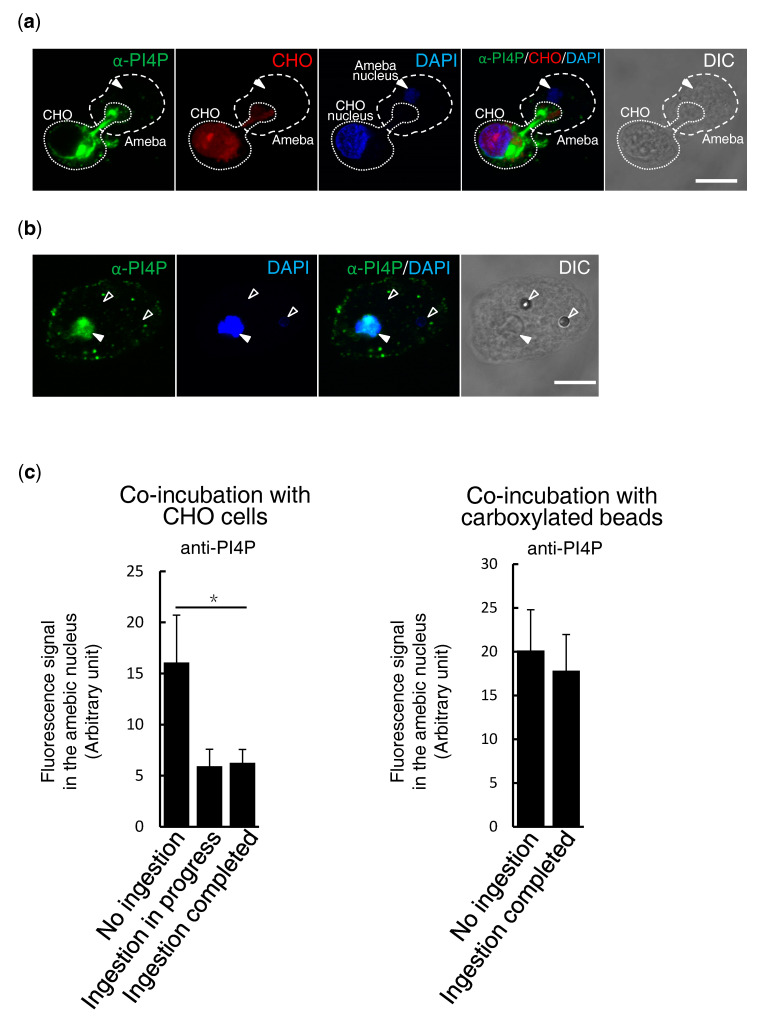
Elimination of nuclear PI4P by the ingestion of CHO cells, but not beads. (**a**,**b**) Immunofluorescence imaging of PI4P during trogocytosis of live CHO cells (**a**) and phagocytosis of carboxylated beads (**b**). PI4P and nuclei were visualized by anti-PI4P antibodies (green) and DAPI (blue), respectively. (**a**) *E. histolytica* trophozoites were co-cultured with CHO cells, prestained with CellTracker Red, for 10 min at 35.5 °C. White solid arrowheads indicate the nuclei of *E. histolytica* trophozoites. The broken and dotted lines depict the edge of the ameba and CHO cell, respectively. (**b**) *E. histolytica* trophozoites were co-cultured with carboxylated beads at 35.5 °C for 4 h. White solid arrowheads indicate the nuclei of *E. histolytica* trophozoites. Open arrowheads indicate ingested carboxylated beads. (**c**) Quantitation of PI4P in the nucleus. The fluorescence intensity in the nucleus of the trophozoites incubated with CHO cells (left panel, *n* = 8) or carboxylated beads (right panel, *n* = 15) was measured and is shown in arbitrary units. Amebae were categorized into two or three groups: those which did not ingest CHO cells or beads (“No ingestion”), those in the process of ingesting CHO cells (“Ingestion in progress”), and those in which the ingestion of CHO cells or beads was completed (“Ingestion completed”). Scale bar = 10 µm. Statistically significant differences with *p* < 0.05 by Student’s t-test is depicted with “*”.

To understand whether the decrease of PI4P (most likely by metabolism or translocation) in the nucleus is dependent on prey, *E. histolytica* trophozoites were co-cultured with CellTracker Red-stained live CHO cells or carboxylated beads, and PI4P was detected by anti-PI4P antibodies. After co-culture with live CHO cells to allow trogocytosis, PI4P was decreased in the nucleus (*p* < 0.05; Figure 3a,c). However, PI4P localization was not affected by the co-culture with carboxylated beads (Figure 3b,c), suggesting that elimination of PI4P from the nucleus occurred only when mammalian cells were given to the amebae.

### 3.4. PI4P Elimination fro Amebic Nuclei is Triggered by Human Erythrocyte Membrane Fraction

**Figure 4:**
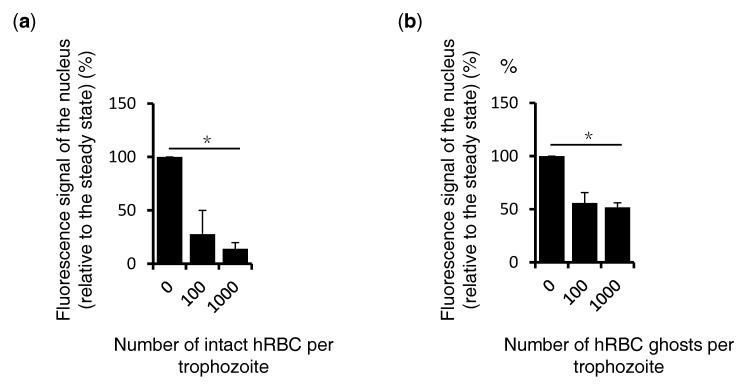
Quantification of the nuclear localized PI4P signal in the ameba by IFA during the phagocytosis of human erythrocytes. *E. histolytica* trophozoites were incubated with whole human erythrocytes (**a**) or their erythrocyte membrane fraction (**b**) for 15 min. After fixation, PI4P was visualized by immunofluorescence imaging with anti-PI4P antibodies. The confocal images were analyzed by Adobe Photoshop as described in the Materials and Methods section. The signal intensity was normalized against that of the control with no erythrocytes. Bars indicate standard errors. The statistical significance was tested by Student’s t test and adjusted by the Bonferroni correction (*p* < 0.05). Statistically significant differences with *p* < 0.05 by Student’s t-test are depicted with “*”.

We further investigated whether the membrane components of erythrocytes are responsible for triggering the observed PI4P dynamism. *E. histolytica* was co-cultured with whole intact erythrocytes or their membrane fraction, and the nuclear PI4P intensity was measured. After the addition of 100 or 1000 intact erythrocytes, the nuclear PI4P signal decreased (Figure 4a), which is consistent with the data shown in Figure 1 and Figure 2. Similarly, the PI4P intensity in the nucleus was decreased after the addition of the membrane fraction of an equivalent number (1000) of erythrocytes (Figure 4b).

### 3.5. Biochemical Quantitation of Amebic PIPs before and after the Addition of the Human Erythrocyte Membrane Fraction

**Figure 5:**
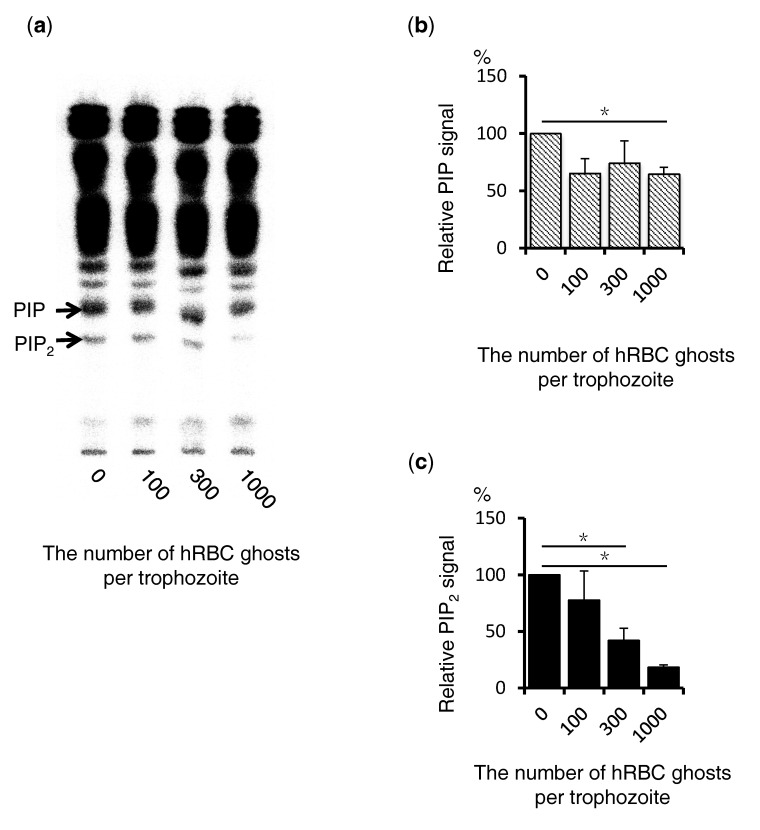
Quantification of PIPs in total cell lysates before and after the phagocytosis of the membrane fraction of human erythrocytes by TLC. (**a**) Amebic trophozoites were metabolically labeled with ^32^P phosphate for 4 h and subsequently incubated with human erythrocytes for 15 min to allow phagocytosis. Lipids were extracted and subjected to TLC. The radioisotope signal on TLC plates was captured and analyzed on Typhoon. The numbers on the bottom axis indicate the number of human erythrocytes equivalent to the membrane fraction used per lane. (**b**,**c**) The radioisotope signal of each band corresponding to PIP and PIP_2_ on the TLC plate, shown in (**a**), was measured by Fiji and is shown in these graphs. The signal values were normalized against those in the control sample without human erythrocytes and shown as a percentage (*n* = 3). Bars depict standard errors. Asterisks’ indicate statistically significant differences with *p* < 0.05 by Student’s t test and adjusted by the Bonferroni correction. Statistically significant differences with *p* < 0.05 by Student’s t-test are depicted with “*”.

To quantify the amount of PIPs, TLC was performed using ^32^P labeled trophozoites. Amebic trophozoites were incubated in ^32^P-phosphate containing BIS medium to label PIPs. Total lipids were extracted by Bligh−Dyer method and developed by TLC. PIP and PIP_2_ were identified based on the Rf values of standards (Figure 5a). The signal intensity of PIP appears to be reduced after the addition of the erythrocyte membrane fraction (Figure 5b). Likewise, the PIP_2_ intensity was also significantly decreased (*p* < 0.05 (Figure 5c)).

### 3.6. Immunofluorescence Imaging Showing the Localization of PI(4,5)P_2_ in Steady State and during Erythrophagocytosis

**Figure 6:**
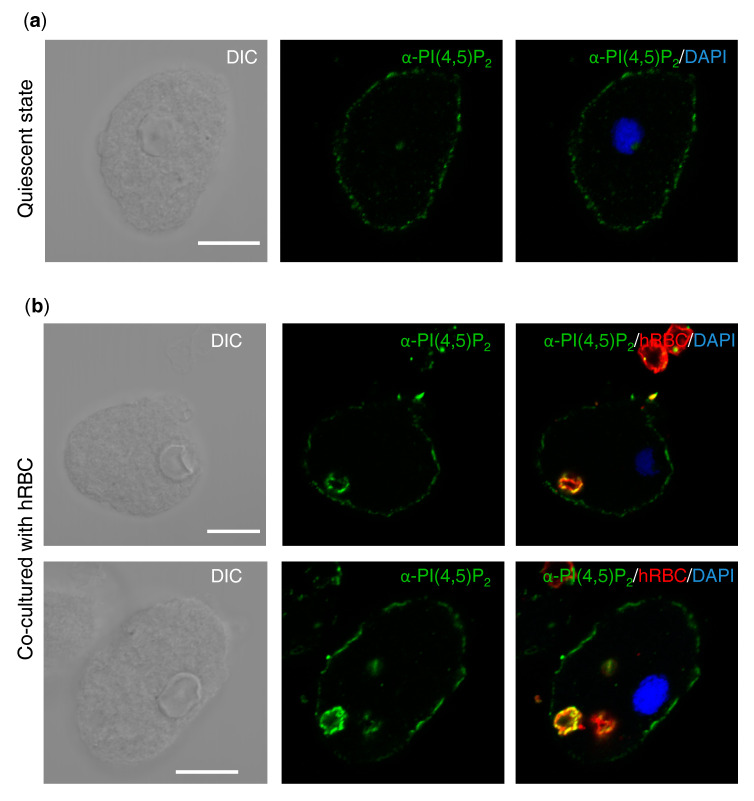
PI(4,5)P_2_ localization in *E. histolytica* trophozoites in steady state (**a**) and during the phagocytosis of human erythrocytes (**b**). PI(4,5)P_2_ in *E. histolytica* trophozoites in steady state (**a**) or during the phagocytosis of human erythrocytes, which were prestained with PKH26 (**b**) was visualized by anti-PI(4,5)_2_ antibodies conjugated with fluorescein (green). Nuclei in amebic trophozoites were stained by DAPI (blue). Scale bar = 10 µm.

To better understand the fate of PI4P in the nucleus upon erythrophagocytosis, PI(4,5)P_2_ was visualized with anti-PI(4,5)P_2_ antibodies. PI(4,5)P_2_ is the major species of PIP_2_ and is mostly localized on the plasma membrane. PI(4,5)P_2_ can be metabolized to other PIPs or inositol 3-phosphate (IP_3_) and diacylglycerol (DAG). The quantitation of PIP_2_ by TLC indicated, as shown above, that the amount of PIP_2_ was reduced upon erythrophagocytosis. Immunofluorescence imaging showed that in a quiescent state, PI(4,5)P_2_ was localized on the plasma membrane of all (100%) *E. histolytica* trophozoites (Figure 6a). After the addition of erythrocytes prestained with PKH26, the plasma membrane localization and signal intensity of PI(4,5)P_2_ were apparently unchanged (Figure 6b). Neither was PI(4,5)P_2_ observed in the nucleus. Therefore, the elimination of PI4P from the nucleus upon interaction with human erythrocytes is not attributable to the in situ formation of PI(4,5)P_2_.

## 4. Discussion

### 4.1. The First Case of Visualization of Nuclear PI4P by Antibody or Biosensor

It is known that PI3P, PI4P, PI5P, PI(4,5)P_2_, and PI(3,4,5)P_3_ are localized in the nucleus. Nuclear PIPs were previously only detected by TLC or HPLC via radioisotopic labeling. While PI(4,5)P_2_, the most abundant PIP_2_ in cells, has been demonstrated in the nucleus by IFA or immunoelectron microscopy using anti-PI(4,5)P_2_ antibodies [13], the demonstration of PI4P in the nucleus by imaging has been challenging and, thus, has never been reported previously, unlike PI3P or other phospholipids. The pleckstrin-homology (PH) domain has been used as the most popular PI4P biosensor; however, concerns on the cross specificity of this domain toward PI4P and PI(4,5)P_2_ is well known [14]. SidM, which was recently used as a monomer or two tandem copies for PI4 biosensing [12], showed monospecificity toward PI4P, and was used to demonstrate PI4P localization on the plasma membrane endosomes and lysosomes. In this report, we have demonstrated, for the first time in eukaryotes, the nuclear localization of PI4P in *E. histolytica* by IFA and immunoelectron microscopy using anti-PI4P antiboies and SidM domain. This is the first example of visualization of nuclear localized PI4P in eukaryotes.

### 4.2. The Reduction of Nuclear PI4P upon Mammalian Cell Attachment can be Mediated by Metabolism or the Transport to the Cytoplasm

In this study, we demonstrated the reduction of nuclear PI4P by IFA and immunoelectron microscopy, and the reduction of the cellular PIP_2_ level by TLC, upon mammalian cell attachment. There are a few possible explanations for the observed nuclear PI4P reduction, e.g., metabolism in the nucleus and transport to the cytosol. Nuclear PI4P can be metabolized to PI, PI(3,4)P_2_, or PI(4,5)P_2_. However, the two former metabolites are unlikely to be produced from PI4P because neither PI4-phosphatase nor Type II PI3-kinase is present in the *E. histolytica* genome. On the contrary, PI(4,5)P_2_ can be synthesized from PI4P, as *E. histolytica* has Type II PI5-kinase (EHI_153770), which contains a potential nuclear localization signal (NLS) [15]. PI(4,5)P_2_ is further metabolized to DAG and inositol 3-phosphate (IP_3_) by phospholipase C (PLC). IP_3_ works as a trigger of Ca^2+^ release and is involved in cytoskeletal remodeling [16], and is thus likely involved in erythrophagocytosis where Ca^2+^-dependent cytoskeletal reorganization plays a major role [17,18]. It was previously reported that *E. histolytica* has an IP_3_ and IP_4_-dependent Ca^2+^ release to the cytosol [19]. *E. histolytica* possesses 27 calcium binding proteins, some of which were shown to be involved in the regulation of the cytoskeleton during phagocytosis. Another fate of PI(4,5)P_2_ may be PI(3,4,5)P_3_. *E. histolytica* has four inositol polyphosphate multikinases (IMPKs) [20], potentially involved in the nuclear synthesis of PI(3,4,5)P_3_ [21]. PI(3,4,5)P_3_ is known to bind and activate steroidogenic factor 1 (SF1) in other organisms [22]. Interestingly, one of *E. histolytica* IMPKs possesses a nuclear localization signal, but an SF1 homologue is missing in the genome. It is still possible that in *E. histolytica*, a PI(3,4,5)P_3_-mediated transcriptional regulation is operated with a functional homologue of SF1.

Another scenario to explain the nuclear PI4P reduction induced by the attachment of mammalian cells is the nucleus-to-cytoplasm transport of PI4P by lipid transfer proteins (LTPs). *E. histolytica* has two lipid transfer proteins which have NLS [23]. It is plausible that these NLS-containing LTPs possibly bind to PI4P, and are involved in PI4P translocation to the site of action in the cytosol or the presentation of phosphatidylinositol (PI) to the PI4P-producing enzyme(s) upon the erythrocyte’s attachment to the ameba. To further elucidate the localization, metabolism, and traffic of PIPs, each PIP species in individual compartments needs to be detected and quantitated.

### 4.3. The Reduction of PI4P and PI3P is Target Dependent

The reduction of PI4P from the nucleus was observed only when live erythrocytes, its membrane ghost, or live CHO cells were used as prey, whereas artificial beads did not induce nuclear PI4P reduction. A similar dependence on the prey was previously demonstrated for PI3P recruitment to phagosomes. GFP-HrsFYVE, a PI3P bioprobe, was recruited to phagosomes that contained serum-coated beads, but not to those containing non-coated beads in *E. histolytica* [3,6]. Furthermore, it was demonstrated that the trogocytosis of CHO was enhanced while the phagocytosis of non-coated beads was repressed by HrsGFP-FYVE overexpression [3], suggesting that the role of PI3P may differ in live cell trogocytosis and bead phagocytosis. Taken together, the metabolism and/or translocation of PI3P and PI4P is apparently target dependent, which may elicit different signaling pathways.

## Supplementary Materials

The following are available online at https://www.mdpi.com/2076-2607/8/7/1050/s1, Figure S1: Establishment of the myc-SidM expressing strain. Figure S2: Representative images of immunoelectron microscopy using anti-PI4P antibody.
