# Supplementary material for: Dynamism of PI4-Phosphate during Interactions with Human Erythrocytes in Entamoeba histolytica
Source: Microorganisms. 2020 Jul 15;8(7):1050. doi: 10.3390/microorganisms8071050 (PMC7409237; doi:10.3390/microorganisms8071050)
Supplement: Supplementary file 1 [file microorganisms-08-01050-s001.pdf]

microorganisms

Supplementary Information

Dynamism of PI4-phosphate during interaction with human erythrocytes in *Entamoeba histolytica*

Natsuki Watanabe[1], Kumiko Nakada-Tsukui[2], Tomohiko Maehama[3], Tomoyoshi Nozaki[1,*].

[1] Department of Biomedical Chemistry, Graduate School of Medicine, The University of Tokyo.
[2] Department of Parasitology, National Institute of Infectious Diseases, Tokyo, Japan.
[3] Division of Molecular and Cellular Biology, Graduate School of Medicine, Kobe University, Kobe, Japan.
* Correspondence addressed to: Tomoyoshi Nozaki, 7-3-1 Hongo, Bunkyo-ku, Tokyo 113-0033, Department of Biomedical Chemistry, Graduate School of Medicine, The University of Tokyo; e mail, nozaki@m.u-tokyo.ac.jp; tel, +81-3-5841-3526

Received: date; Accepted: date; Published: date

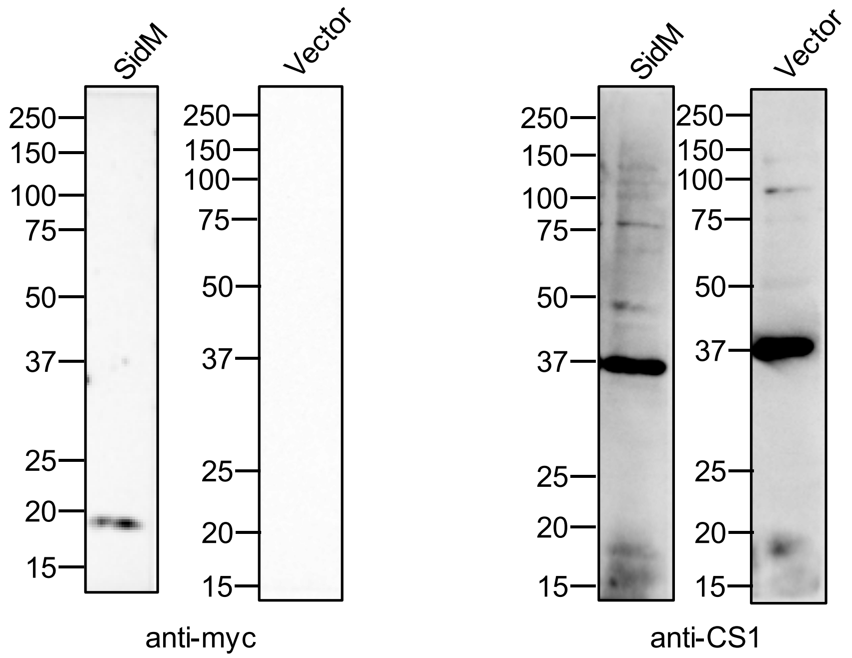

Figure S1. Establishment of the myc-SidM expressing strain. Expression of myc-SidM was detected by immunoblot using anti-myc antibody (left panel). Cysteine synthase 1 (CS1) was used as a loading control and was detected using anti-CS1 antiserum (right panel). A transformant transfected with an pEhEx-myc empty plasmid (“Vector”) was also used as control.

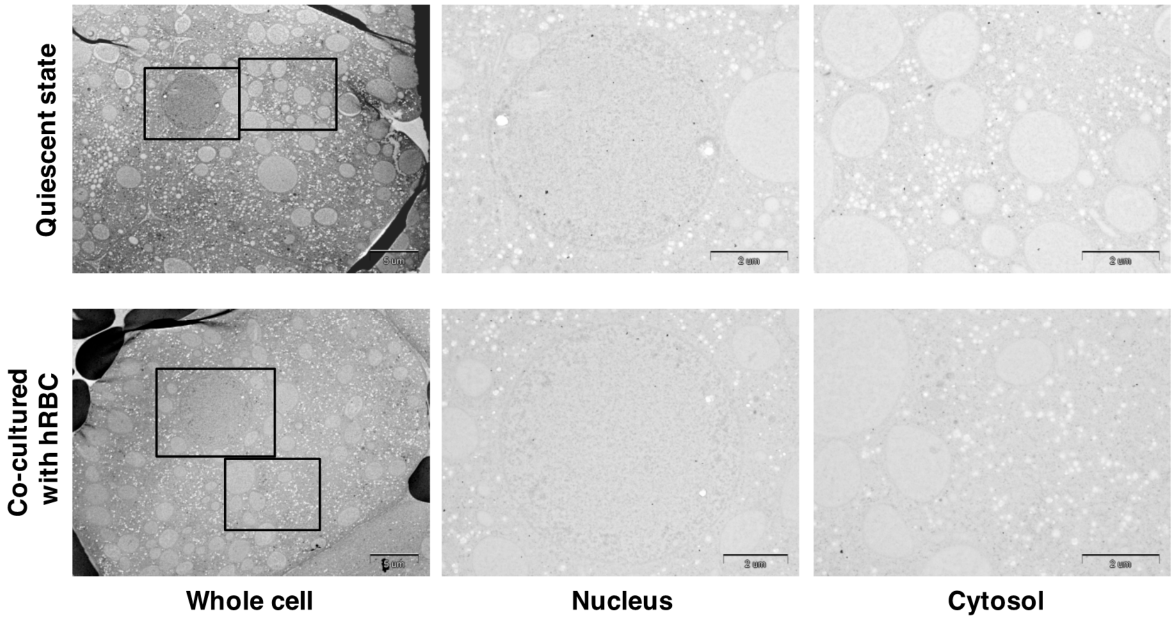

Figure S2. Representative images of immunoelectron microscopy of amebic trophozoites in the absence or presence of human erythrocytes. Localization of PI4P was detected using anti-PI4P antibody and 10 nm gold anti-mouse secondary antibody. Signals localized in the nucleus were counted and presented in Figure 2. Black rectangles were magnified regions of the nucleus and cytosol.

© 2020 by the authors. Submitted for possible open access publication under the terms and conditions of the Creative Commons Attribution (CC BY) license (http://creativecommons.org/licenses/by/4.0/).
